# Effectiveness of flipped classroom pedagogy: A mixed-methods analysis of adult continuing education—Case study, Egyptian context

**DOI:** 10.1371/journal.pone.0353699

**Published:** 2026-07-16

**Authors:** Hala Mohamed Said, Islam Al Sawi, Heba Alaa-Eldeen Said

**Affiliations:** 1 Academy of Liberal Arts, American University in Cairo, Egypt; 2 University of Nizwa, Department of Foreign Languages, English Section, Oman; 3 School of Continuing Education, American University in Cairo, Egypt; Hong Kong Metropolitan University, HONG KONG

## Abstract

This mixed-methods study investigated the effectiveness of a flipped classroom model in an adult English as a Foreign Language (EFL) continuing education program in Egypt. A total of 297 learners participated by responding to an online questionnaire, yielding a 72.6% response rate. Learner perceptions were assessed using a Model for Improving Student Learning (MISL) framework across five instructional dimensions, demonstrating strong internal consistency (Cronbach’s α = .72–.93). Quantitative findings indicated positive perceptions across all dimensions (mean scores: 4.30–4.52 on a 5-point scale), with Knowledge Construction rating highest. Multiple regression analysis (R² = .668) identified learner confidence, motivation, engagement, and digital competence as significant predictors of perceived learning. A multivariate analysis (MANOVA) found no significant effect of prior flipped classroom experience. Thematic analysis of open-ended responses revealed increased participation, confidence, and collaboration. The study extends conventional mixed-methods analysis by incorporating sentiment analysis of qualitative responses using Python’s TextBlob library. Sentiment analysis supported these results, with overall positive polarity scores. Findings underscore the role of affective and digital readiness factors in flipped classroom effectiveness, emphasizing the critical importance of well-structured implementation over prior experience with the flipped classroom model, with implications for curriculum design in technology-enhanced adult education, especially in non-Western, resource-constrained settings.

## 1. Introduction

The flipped classroom model, which restructures traditional lecture-based instruction by shifting direct content delivery outside of class and using in-class time for active, collaborative learning, has received increasing attention in education worldwide [1–5]. Grounded in constructivist learning theory, the flipped classroom approach enables higher-order cognitive engagement and self-directed learning within learners’ zones of proximal development (ZPD) [6,7]. While numerous studies report benefits across disciplines, research reveals that outcomes depend heavily on cultural context, implementation quality, and learner characteristics, with effectiveness varying significantly based on local educational environments and student demographics [4,8].

Despite extensive global research, critical gaps persist in understanding flipped classroom effectiveness within specific contexts. As Little [9] documented, there remains a notable absence of published research on flipped classroom implementation in post-compulsory education, a gap that persists to this day and extends well beyond any single geographic region. Current research remains predominantly focused on younger or full-time student populations, with limited systematic attention to adult or continuing education EFL/ESL settings worldwide [10]. This demographic limitation represents a significant challenge in flipped classroom research, as adult learners present unique characteristics and learning needs that may not be adequately addressed by research conducted with traditional compulsory education, a gap that this research aims to address.

Given the persistence of this global gap, exploring particular contexts, such as Egyptian EFL education, offers insights that may be relevant to diverse educational contexts facing similar challenges. Research in Egyptian EFL contexts [11–14], while providing valuable insights into flipped classroom potential, has concentrated on discrete language skills rather than holistic learning experiences. These Egyptian EFL studies have typically targeted specific linguistic domains such as writing performance, morphological affixes, grammatical accuracy, and listening comprehension, often measuring short-term academic outcomes rather than broader learner experiences. However, recent research emphasizes that flipped classroom success depends not merely on discrete language skill development but critically on affective and metacognitive factors, particularly learner confidence and self-efficacy [15,16]. These factors may function differently among adult populations who are balancing educational goals with professional and personal responsibilities. For adult EFL learners in continuing education, where voluntary participation and immediate practical language goals intersect with diverse technological backgrounds and varied prior educational experiences, understanding which factors most influence learning satisfaction becomes essential for both pedagogical effectiveness and institutional sustainability, another gap that this study aims to address.

Situated within an Egyptian university’s continuing education program, this study thus addresses these persistent research gaps by examining how adult EFL learners perceive and experience flipped classroom instruction. Building on established constructivist frameworks and employing mixed-methods analysis, the research investigates not only overall perceptions of effectiveness but also the interplay between learner characteristics, contextual factors, and perceived learning outcomes. By examining both quantitative patterns and individual learner voices, this study aims to inform evidence-based curriculum design for adult EFL continuing education and contribute to the underexplored intersection of flipped pedagogy and adult language learning.

### Research questions

1. How do adult students in a continuing education program perceive the flipped classroom experience in general English instruction across the five dimensions of Awidi’s [17,18] constructivist framework?
  1. Which learner characteristics (digital competence, learner confidence, personal motivation, and personal engagement) significantly predict perceived learning outcomes in an adult, continuing education EFL flipped classroom setting?
  2. Does prior experience with flipped learning significantly influence adult students’ perceptions of their current flipped EFL classroom experience in a continuing education program?
  3. What themes emerge from the open-ended reflections of adult students in a continuing education program on how pre-class preparation and flipped learning influence their participation, engagement, and learning?
  4. What is the overall sentiment of the reflections of adult students in a continuing education program on the flipped classroom evidence, and how does this sentiment vary across different aspects of the learning process?

## 2. Review of literature

### 2.1 Flipped classroom: Frameworks and implementation across disciplines

The flipped classroom, also known as flipped learning or the inverted classroom, is a blended learning strategy that restructures the traditional teaching process by delivering core instructional content outside formal class meetings (typically via videos, readings, or online modules), thereby freeing up classroom time for active, collaborative, and higher-order learning experiences. This approach, popularized by Bergmann and Sams [6] and Bishop and Verleger [7], represents an expansion, not a simple reshuffling, of the curriculum, permitting richer, more flexible engagement with both content and analytical tasks.

Flipped learning draws from constructivist and socio-constructivist theory: learning is seen as an active process of knowledge construction, best supported by scaffolding and social interaction [19]. The model enables students to prepare independently at their own pace, then participate in group-based, instructor-guided tasks within their “zone of proximal development.” By focusing in-person time on practice, discussion, and feedback-rich activities, flipped learning aligns with Bloom’s taxonomy in emphasizing higher-order cognitive skills [7,20].

Building on the emphasis on learner autonomy and collaborative participation introduced in the previous paragraph, Awidi’s [17,18] Model for Improving Student Learning (MISL) provides a comprehensive theoretical framework for flipped classroom pedagogy, particularly suited for adult learners in continuing education settings. The MISL model identifies five key dimensions: Access to Information and Resources (AIR), Support and Motivation (SAM), Participation in Learning Activities (PAC), Assessment and Feedback (ASF), and Knowledge Construction (KCO), which align closely with Knowles’ principles emphasizing self-directed learning, prior experience, and intrinsic motivation in adult learners [21,22]. Knowles [21] emphasizes empowerment as critical in supporting adult development within organizational contexts. From a psychological perspective, Awidi’s MISL integrates Bandura’s [23] self-efficacy theory by embedding mechanisms that build learner confidence and motivation through structured support and active engagement. Notably, the SAM and PAC dimensions correspond with self-efficacy concepts, emphasizing motivational support and meaningful participation as drivers of self-regulated learning. Thus, Awidi’s MISL dimensions theoretically align with the key constructs of confidence, motivation, and self-regulation in adult learning and educational psychology scholarship by Knowles [21,22] and Bandura [23]. This alignment provides a clear theoretical lens for understanding and implementing effective flipped classroom pedagogies in adult continuing education contexts.

Beyond this theoretical foundation for adult education, research shows flipped classroom adoption across STEM, health, and language fields yields divergent evidence regarding skill development. For instance, Shao & Liu [8] found that while the flipped classroom can increase engagement and academic performance compared to traditional instruction, outcomes vary considerably based on student demographics, implementation quality, and the skills targeted. Similarly, Pongpanich et al. [4] report that context and culture may greatly impact the success of the model. Thus, to effectively maximize the benefits of the flipped classroom and ensure its success, literature surveys such as these highlight the importance of culturally and contextually informed adaptation, highlighting that progress depends on careful consideration of local attitudes, technological infrastructure, and institutional support.

Consistent with this emphasis on context and culture, in the Middle East and North Africa (MENA), and Egypt in particular, a modest but growing body of research has started to document systematic evidence for skill development across disciplinary contexts. For example, Kakarougkas & Abdellatif [24] implemented a flipped classroom model in an undergraduate molecular biology course at the American University in Cairo. Students generally reported high engagement and improved information retention; however, the study noted that workload and time management emerged as barriers and warned against adopting the model in multiple courses simultaneously due to the increased demand on students’ time. The investigation was limited by a small cohort (n = 15) and discipline specificity. Similar patterns emerged from research on blended/flipped biology classes during COVID-19 [25] and on the use of the flipped model in practical parasitology [26], where gains in engagement, satisfaction, and academic achievement were accompanied by practical and contextual obstacles, including constraints on access to online resources, small sample sizes, and institutional specificity.

### 2.2 Flipped classroom in the context of EFL

The flipped classroom’s integration of self-paced content delivery, collaborative practice, and scaffolded support offers unique advantages for language learning contexts. This foundation sets the stage for a closer examination of how flipped learning has been applied to English as a Foreign Language (EFL) skill development.

In his systematic review, Arslan [15] screened 78 peer-reviewed studies from major research databases, prioritizing quality and robust methodology. He reports that research on flipped learning in EFL/ESL settings concentrates on university student populations and targets discrete skills like writing and speaking, demonstrating clear benefits for language skill development. Several findings report that this approach positively influences reading comprehension [27], speaking skills [28], and listening proficiency [29].

A handful of Egyptian studies have investigated the flipped classroom’s effect on EFL subskills such as writing, grammar, and morphology with different designs and scopes. In a recent study, Abdelghafar et al. [11] implemented a flipped WebQuest model to enhance English writing among secondary school students. The results showed that students in the experimental group not only showed significantly improved writing performance but also experienced lower L2 writing anxiety compared to those taught with traditional methods. Notably, the intervention fostered greater learner autonomy and more frequent peer collaboration.

Similarly, Marghany [14] examined a flipped classroom-based program targeting the mastery of morphological affixes among Egyptian EFL sophomores. The experimental group outperformed the control, confirming short-term effectiveness in this specific linguistic domain. The study nonetheless acknowledged contradictory findings in prior work and warned against assuming transferability to other EFL skills or contexts.

Desouky [30] conducted a controlled university study to assess the impact of a flipped learning approach on general English proficiency among Egyptian EFL undergraduates. The study split students into experimental and control groups, comparing a flipped classroom model with traditional communicative instruction over two semesters. While the flipped group demonstrated a statistically significant advantage only on final exam scores, surveys and teacher observations revealed greater engagement, enthusiasm, and learner autonomy among these students throughout the intervention. Importantly, students reported higher satisfaction and increased participation in flipped classes.

Research by El-Bassuony [13] focused on English grammatical performance among underachieving secondary students. The research utilized both control and experimental groups to compare the effectiveness of flipped learning versus traditional instruction. Participants included underachieving students alongside their peers at the same educational level. The flipped model led to statistically significant improvements in grammar use during both speaking and writing tasks for underachieving learners and their peers. Individualized attention, peer support, and multiple practice opportunities were highlighted as key benefits. Even so, the study noted several limitations, including the relatively small sample size, the specific characteristics of the sample, the limited range of grammatical rules targeted, and the length of the intervention.

Other Egyptian research has explored the effects of technology-enhanced methods on EFL receptive skills, though significant methodological limitations remain. Ahmad [12] investigated the effect of the flipped classroom model on listening comprehension among a cohort of 34 third-year EFL students at the Faculty of Education, Suez University. The study employed a one-group pre-post design assessing listening comprehension before and after exposure to the flipped classroom intervention. The study reported statistically significant improvements in listening scores following the intervention.

Recent literature reviews and meta-analyses, however, emphasize that the flipped classroom’s value extends well beyond discrete language skills, with evidence of substantial effects on affective and metacognitive aspects of learning, notably, motivation, confidence, engagement, and shifts in learner mindset [8,15,31]

Among these affective dimensions, learner confidence emerges as particularly critical. The theoretical foundations for understanding learner confidence in flipped environments can be traced to Bandura’s [26] work on self-efficacy: individuals’ beliefs in their capabilities to perform specific tasks. Self-efficacy significantly influences motivation, persistence, and learning outcomes, making it particularly critical in flipped classrooms where learners assume greater responsibility for self-directed preparation and active participation. Empirically supporting this perspective, Yilmaz [16] conducted a study with 178 undergraduate students to assess their e-learning readiness and satisfaction with flipped classroom instruction. Using an e-learning readiness scale (ELRS) that measured technological preparedness and self-efficacy in e-learning environments, the study found a positive correlation between readiness scores and student satisfaction and motivation in flipped classrooms. Critically, Yilmaz [16] demonstrated that students’ confidence in navigating e-learning environments (a form of self-efficacy) was a stronger predictor of success than mere technical computer skills. These results reinforce the emphasis on affective factors over technical competence in technology-enhanced learning environments.

Building on this understanding, many empirical studies have investigated the factors that contribute to the success of the flipped classroom model. Among those that are most commonly reported are learners’ attitude, self-regulation, teacher training, technological access, digital literacy, and purposeful instructional design [3,10,15].

However, the literature on the impact of prior familiarity with flipped classrooms is notably inconclusive. Lo and Hew [20] found that unfamiliarity could cause early resistance or disengagement in K-12 learners but also observed that well-planned onboarding and support often mitigated these effects. Shao and Liu’s [8] meta-analysis, spanning K-12 and university settings, found inconsistent links between prior experience and outcomes. Likewise, Lestari [32] observed that novice pre-service EFL teachers, with effective scaffolding, matched the satisfaction and engagement of experienced peers in flipped classroom settings. These studies suggest that while prior familiarity may affect early adaptation, it does not reliably determine satisfaction or outcomes, highlighting the need for research in adult continuing education contexts [8,20,32].

### 2.3 Flipped classroom and persistent gaps in the literature

Evidence supports that active, collaborative flipped designs boost participation and willingness to communicate [4,31], while digital access and resource equity influence engagement [10,15]. Constructive feedback, whether digital or in-person, underpins skill development and learner autonomy [3,8]. Yet, there is no consensus on which elements most influence satisfaction or learning, and studies rarely investigate combinations of these dimensions [3,4,8,10,31].

Additionally, there is a clear lack of sufficient published studies and documented implementation of flipped classroom approaches in adult education contexts. Little [9] concludes that there were no published papers on the implementation of the flipped classroom model in post-compulsory education in the UK. This significant gap in the literature seems to persist up until now and well beyond the UK. Recent meta-analyses and surveys of the literature continue to report that traditional teaching methods dominate continuing professional development and lifelong learning activities [3,33]. This may be due to a significant gap in understanding how flipped instruction affects adult learners, who have unique cognitive, motivational, and experiential traits [9,10].

Current research remains predominantly focused on younger or full-time student populations, with limited systematic attention to adult or continuing education EFL/ESL settings [10]. The recent review by Pongpanich et al. [4] similarly finds that research concentrates primarily on higher education, with little attention given to other settings. In Egypt, far less is known about the learning experience of adult or continuing education EFL learners in flipped learning environments. A more nuanced understanding of how flipped models impact broader educational outcomes remains limited, particularly in non-compulsory settings.

Altogether, literature affirms the relevance of engagement, access, participation, and feedback for flipped classroom effectiveness but lacks empirical clarity on which factors matter most or how prior experience shapes outcomes for EFL learners, particularly in the context of adult EFL non-traditional or voluntary education settings. This clear gap justifies the present study, which aims to disentangle these influences in a more focused investigation into EFL flipped learning specifically tailored for adult continuing education settings.

Overall, this review demonstrates that the field can still benefit from robust research that systematically investigates the wider educational impacts of flipped learning on adult EFL learners in Egypt, which is precisely the aim of the current investigation. Given these gaps, it is imperative to examine how the flipped classroom mediates engagement, achievement, and motivation in a continuing education adult EFL context.

## 3. Methodology

### 3.1 Theoretical framework

This study employed Awidi’s [17,18] constructivist scaffolding framework to evaluate adult learners’ perceptions of the flipped classroom learning experience in an English as a Foreign Language (EFL) continuing education context. The five-dimensional Model for Improving Student Learning (MISL), encompassing Access to Information and Resources (AIR), Support and Motivation (SAM), Participation in Learning Activities (PAC), Assessment and Feedback (ASF), and Knowledge Construction (KCO), provides a holistic evaluation approach that addresses both technological and pedagogical aspects of digital learning [18]. The framework’s theoretical grounding in social constructivism aligns with communicative language teaching principles fundamental to effective EFL instruction. Originally developed within African higher education contexts (Ghana), its suitability for the Egyptian educational context stems from shared challenges related to technology integration and pedagogical transformation. The framework’s applicability has been supported by subsequent studies, including Awidi and Paynter’s [34] successful application in flipped classroom evaluation, demonstrating its relevance for technology-enhanced learning environments.

### 3.2 Institutional context and course design

The research was conducted at the American University in Cairo (AUC) at the School of Continuing Education (SCE), Languages Department, which offers a comprehensive English language program comprising 16 General English levels and nine Conversational English levels designed for adult learners of varied ages and educational backgrounds. At the SCE, courses are structured in six-week terms, with multiple sections per level each semester, ranging from 10 to 20 sections depending on enrollment demand. Access to program details can be accessed via this link: https://sce.aucegypt.edu/programs/english-courses

The Languages Department at the SCE is currently undertaking a comprehensive redesign project aimed at fully aligning curriculum and instructional delivery with flipped classroom principles. This initiative seeks to standardize a well-designed flipped classroom model across nearly all 25 program levels. At the institution, both the A1 beginner stage and the A2 elementary stage are each subdivided into four progressive levels. As of this study, the redesigned flipped classroom approach has been developed and launched for eight general English course levels, spanning the complete A1 and A2 stages (A1.A through A2.D), covering the first year of the project. The A1D course referenced in this study corresponds to the final level of the A1 beginner stage.

The redesigned courses implement a flipped, task-based pedagogical framework structured around two integrated learning phases. This design corresponds to widely established flipped classroom models documented in educational research, particularly the foundational frameworks developed by Bergmann and Sams [6] and Bishop and Verleger [7], and aligns with the empirically derived design principles documented by Kim et al. [35] in their study of multiple flipped classroom implementations. The framework incorporates key factors identified in comprehensive research on successful flipped classroom implementation while being specifically adapted to the contextual requirements of adult EFL continuing education, available technological resources, and the diverse learning needs of Egyptian learners.

#### 3.2.1 Pre-class preparation (Moodle platform).

Students independently access the learning management system, Moodle, which hosts all essential course information, including syllabi, learning objectives, and assessment schedules, as well as instructional content for 12 sessions per course. The pre-class phase requires an average of three to four hours weekly of engagement with carefully sequenced instructional materials focusing on receptive skills and language subskills. These materials include interactive videos, listening and reading activities, grammar tutorials, and vocabulary and writing lessons, all designed with immediate feedback mechanisms to support autonomous learning. The Moodle instructional material and interactive activities are purposely designed to be multimodal, scaffolded, and responsive to diverse learning preferences and proficiency levels. Structured reflection activities are incorporated in the design of the majority of the sessions on Moodle to encourage students to evaluate their progress and identify areas requiring clarification before attending face-to-face sessions.

#### 3.2.2 Face-to-face sessions (classroom component).

Students attend two face-to-face sessions per week, each lasting 2.5 hours. Classroom time focuses on collaborative speaking and writing tasks that build upon pre-class preparation. Students engage in peer and group tasks involving negotiation of meaning, collaborative problem-solving, communicative practice, and reciprocal feedback. Teachers facilitate these interactions while providing formative feedback on student performance and addressing common errors. Each in-class session begins with a collaborative review of the Moodle preparation experiences and concludes with a reflective exit ticket designed to consolidate learning and promote metacognitive awareness of the entire learning process.

#### 3.2.3 Assessment integration.

Continuous assessment is embedded throughout the course via formative evaluations integrated within Moodle activities, providing ongoing feedback during students’ pre-class preparation. Additionally, both formative and summative assessments are conducted during four designated in-class sessions, aligning closely with learning objectives and classroom activities to comprehensively monitor and support student progress.

### 3.3 Sampling process and data collection

The study targeted all adult learners enrolled in the A1D course, the final level in the beginner stage, across all sections in a single semester following the implementation of the new design. Including all course sections allowed for capturing instructor and classroom variations, increasing the robustness of findings [36]. From a total of 409 enrolled students, 297 submitted complete and valid responses, yielding a response rate of approximately 72.6%. The study aims to generate insights to guide further adaptation and integration of the flipped model across other course levels in the program. Ethical approval for this research was granted by the institutional review board **(IRB approval #2024-2025-278)**.

The data collection instrument was a secure online questionnaire accessible via a link and a QR code. The questionnaire was adapted from Awidi and Paynter [34], incorporating established items aligned with the MISL framework to assess student perceptions across five dimensions. Items were contextually refined and linguistically adapted for the target adult EFL context in Egyptian continuing education to enhance content validity. To maximize accessibility and response accuracy, the questionnaire was translated into Arabic following standard translation procedures, allowing participants to access and complete the instrument in either English or Arabic according to their preference [37]. This bilingual approach aimed to minimize language barriers and enhance comprehension, thereby improving the validity of participant responses. All instructors teaching A1D were invited to support data collection by sharing the questionnaire with their students during regularly scheduled face-to-face sessions. Several instructors agreed and actively facilitated student participation. The questionnaire was administered by the instructors midway through the six-week term to allow students adequate exposure to both Moodle-based preparation and classroom components. Clear verbal and written guidance accompanied the questionnaire, emphasizing both the voluntary nature of participation and the requirement that participants be at least 18 years old.

The questionnaire comprised 22 closed-ended statements and three open-ended questions. Participants rated their level of agreement with 21 of the statements using a five-point Likert scale ranging from “strongly disagree” to “strongly agree.” 17 of these statements were specifically designed to measure the extent to which students perceived the flipped classroom approach as supporting their learning, aligned with the five key dimensions of MISL [17,18]. Four additional Likert-scale items focused on students’ self-evaluations of their confidence, motivation, and engagement in the course, and their proficiency with digital technologies. The final closed-ended item diverged from the Likert format, requiring a Yes/No response to indicate whether students had previously participated in a flipped classroom. The open-ended questions elicited reflections on how pre-class preparation influenced in-class participation, notable aspects of the flipped classroom experience, and any additional comments.

### 3.4 Data analysis methodology

This study adopted a convergent parallel mixed-methods design [38], integrating quantitative and qualitative analyses to provide a comprehensive understanding of student perceptions of flipped classroom instruction. Quantitative and qualitative data were analyzed concurrently, with findings integrated during interpretation to examine both cognitive and affective dimensions of the learning experience of the flipped classroom experience in our context. The analysis of the questionnaire data involved descriptive statistics, inferential statistical tests, thematic analysis of open-ended responses, and sentiment analysis. All quantitative analyses were conducted using *IBM SPSS Statistics* (Version 31), while qualitative analyses were supported by coding and visualization tools in Python and Google Colab.

To enhance interpretability of the Likert-scale responses and clarify distribution trends, a member of the research team recoded the original five-point scale into three aggregated categories: ‘disagree’ (combining ‘strongly disagree’ and ‘disagree’), ‘neutral’ (‘neither agree nor disagree’), and ‘agree’ (combining ‘agree’ and ‘strongly agree’). Similarly, the dichotomous Yes/No item assessing prior experience with flipped classrooms was recoded numerically to facilitate analysis.

To evaluate the internal consistency of the questionnaire constructs, Cronbach’s alpha was calculated for each of the five core dimensions derived from Awidi’s [17,18] constructivist framework. The reliability coefficients were as follows in Table 1.

**Table 1:** Reliability coefficients, Cronbach’s alpha.

Section	Score
AIR	α = .929 (Excellent)
SAM	α = .849 (Good)
PAC	α = .847 (Good)
ASF	α = .721 (Acceptable)
KCO	α = .912 (Excellent)

These alpha values indicate that each section demonstrated acceptable to excellent internal consistency, supporting the reliability and coherence of the instrument used. This confirmed the appropriateness of proceeding with further quantitative analyses, including multiple linear regressions and Multivariate Analysis of Variance (MANOVA), to explore relationships between independent variables (confidence, motivation, engagement, digital competence, prior flipped experience) and perceived learning outcomes.

In addition, the open-ended responses were subjected to thematic analysis to identify emergent themes. Two of the researchers independently coded the qualitative data and engaged in consensus discussions until full agreement on the themes was reached. To further explore the affective dimension, sentiment analysis was conducted using the TextBlob library to quantify the polarity of student responses on a scale from –1 (highly negative) to +1 (highly positive). This complementary combination of methods enabled a better interpretation of both the cognitive and affective dimensions of student engagement with the flipped classroom experience.

## 4. Results

### 4.1. Descriptive analysis of students’ experience with the flipped classroom

Descriptive statistics indicated that students held positive perceptions of their flipped classroom experience across the five dimensions of Awidi’s [17,18] Model for Improving Student Learning (MISL): Access to Information and Resources (AIR), Support and Motivation (SAM), Participation in Learning Activities (PAC), Assessment and Feedback (ASF), and Knowledge Construction (KCO). Knowledge Construction, reflecting students’ perceptions of how much they learned and internalized through the flipped classroom, received the highest mean rating (M = 4.52, SD = 0.73). This was closely followed by Support and Motivation (M = 4.49, SD = 0.73) and Assessment and Feedback (M = 4.42, SD = 0.70). Access to Information and Resources and Participation in Learning Activities also received strong positive ratings, with means of 4.44 and 4.30, respectively. The results suggest a moderate level of consensus among participants regarding their positive experiences with the flipped classroom model Table 2.

**Table 2:** Descriptive statistics for MISL dimensions (n = 297).

MISL Dimension	Mean	SD
AIR	4.44	0.79
SAM	4.49	0.73
PAC	4.30	0.90
ASF	4.42	0.70
KCO	4.52	0.73

Frequencies provided further insight into the distribution of responses across the MISL dimensions. Overall, students demonstrated a strong positive perception of the flipped classroom approach across all five MISL dimensions. The highest overall agreement was observed in SAM and KCO, with 87.9% and 87.5% of students agreeing, respectively. AIR and ASF also received high agreement scores, at 86.7% and 85.2%, respectively. PAC showed slightly lower agreement at 80.2%, with a correspondingly higher percentage of neutral and disagreeing responses Table 3.

**Table 3:** Frequency of student response to categorized questionnaire items (n = 297).

Element of MISL/questionnaire item	Disagree %	Neutral %	Agree %
**Access to Information and Learning Resources (AIR)**			
The material on Moodle provided me with the necessary resources to complete the course successfully.	4	10.1	85.9
The material on Moodle helped me understand the topic and learning outcomes for each lesson.	1.7	10.1	88.2
I was provided with the necessary resources to successfully complete the course.	4	10.1	85.9
**Combined score for AIR items**	**3.2**	**10.1**	**86.7**
**Support and Motivation (SAM)**			
The material on Moodle motivated me to participate in the class tasks.	3.4	8.8	87.9
Preparing at home and doing tasks in class is a better learning experience than the traditional experience.	5.7	9.8	84.5
It is easy to use the interactive activities on Moodle.	3.7	6.7	89.6
I enjoyed doing the Moodle interactive activities.	3.4	7.1	89.6
**Combined score for SAM items**	**4**	**8.1**	**87.9**
**Participation in Learning Activities (PAC)**			
I competed with my classmates to get a high score on the Moodle leaderboard.	7.4	15.2	77.4
After preparing at home, I found it easier to participate in the speaking and writing tasks in class.	4.7	9.4	85.9
I felt that each of my groups was adequately prepared to work together in the lecture	7.4	15.2	77.4
**Combined score for PAC items**	**6.5**	**13.3**	**80.2**
**Assessment and Feedback (ASF)**			
It was clearly communicated to me how the class tasks and Moodle activities would be checked and how assessments would be graded.	3	6.4	90.6
I prefer answering practice questions online (on Moodle) to doing several graded quizzes in class.	14.8	14.5	70.7
Preparing the material on Moodle before class helped me do better on the assessments.	3.4	9.1	87.5
The instant feedback on the Moodle activities helped me improve my English language skills.	2	6.1	91.9
**Combined score for ASF items**	**5.8**	**9**	**85.2**
**Knowledge Construction (KCO)**			
Doing the speaking and writing tasks in class is a useful way for me to demonstrate the language I have learned.	3	8.8	88.2
Studying the material on Moodle and participating in the class tasks helped me improve my English language.	1.3	11.4	87.2
The assessment items I completed online improved my understanding in this course	1.3	11.4	87.2
**Combined score for KCO items**	**1.9**	**10.5**	**87.5**
**Independent Variables**			
As a learner, I see myself as competent in using digital technologies.	3.7	11.4	84.8
My experience in this course has made me feel confident as a learner.	4	7.7	88.2
My experience in this course has motivated me to learn more.	3.7	8.1	88.2
My experience in this course has fully engaged me as a learner.	3	5.4	91.6
I have participated before in a course with a flipped approach.	56.2	0	43.8

Note: Percentages have been rounded to the nearest decimal.

Looking more closely at individual items, the highest agreement was recorded for ASF, where 91.9% of students agreed that the instant feedback on Moodle activities helped improve their English skills. Similarly, within the AIR dimension, 88.2% agreed that Moodle materials aided their understanding of topics and learning outcomes. In SAM, students expressed high enjoyment and ease of use for Moodle’s interactive activities, with 89.6% agreement on both items. On the other hand, the items with the highest disagreement included a preference-related statement in ASF, where 14.8% of students disagreed with preferring online practice questions over graded quizzes in class. For PAC, 7.4% disagreed with statements about competing on the Moodle leaderboard and adequacy of group preparation. These item-level insights highlight areas of particular strength and some divergence in student preferences within the flipped classroom experience Table 3.

These findings collectively indicate a high level of satisfaction with the pedagogical model employed and reflect positively on the instructional design of the flipped classroom in the context of adult general English education.

### 4.2. Predictors of perceived learning: Regression analysis

To investigate which student characteristics best predicted perceived learning, a multiple regression analysis was conducted with four independent variables: digital competence, learner confidence, personal motivation, and personal engagement. The dependent variable was the composite measure of Knowledge Construction (KCO_avg), reflecting students’ self-assessed learning through the course. The model was statistically significant, F(4, 292) = 146.87, p < .001, and explained a substantial proportion of the variance in perceived learning (R² = .668, Adj. R² = .663), indicating a strong predictive model.

Each predictor made a significant contribution to the model:

- Confidence emerged as the strongest predictor (β = .327, p < .001), suggesting that students’ self-efficacy beliefs in their learning ability significantly influenced how much they perceived they had learned.
- Motivation (β = .236, p < .001) and engagement (β = .197, p = .001) were also significant, consistent with motivational and affective models of learning, which highlight the importance of intrinsic drive and active involvement.
- Digital competence (β = .162, p < .001) was also a significant predictor, though less strong, suggesting that while familiarity with digital tools is valuable, it plays a secondary role compared to affective and metacognitive factors.

The absence of multicollinearity (all VIF values < 5) confirms the independence of the predictors, strengthening the robustness of the model Table 4.

**Table 4:** Multiple linear regression predicting perceived learning from student characteristics.

Predictor	B	Std. Error	β (Standardized)	t	p	VIF
Intercept	0.960	0.156	–	6.150	<.001	–
Digital Competence	0.137	0.036	0.162	3.786	<.001	1.60
Confidence	0.624	0.275	0.327	4.713	<.001	4.23
Motivation	0.236	0.204	0.237	3.574	<.001	3.85
Engagement	0.259	0.177	0.198	3.311	.001	3.13

Model Summary: R =  .817, R² =  .668, Adjusted R² =  .663, F(4, 292) = 146.85, p <  .001

### 4.3. The role of prior flipped classroom experience: MANOVA findings

To assess whether prior exposure to flipped learning influenced students’ learning experience, a one-way Multivariate Analysis of Variance (MANOVA) was performed with prior flipped learning experience (Yes/No) as the independent variable, and the dependent variables were composite mean scores for the five MISL dimensions: AIR, SAM, PAC, ASF, and KCO.

Preliminary checks indicated a significant result for Box’s M test, suggesting the assumption of homogeneity of covariance matrices was violated. However, given the relatively balanced group sizes (No prior exposure, n = 167; Prior exposure, n = 130), the MANOVA is considered robust to such violations, and the results were interpreted accordingly. The multivariate test did not reveal statistically significant differences between groups across the combined dependent variables, Wilks’ Lambda = 0.983, F(5, 291) = 0.989, p = 0.425, partial η² = 0.017, indicating that prior flipped learning experience did not significantly influence overall student perceptions.

Follow-up univariate ANOVAs were conducted for each MISL dimension to examine potential specific domain differences.

The univariate ANOVA results are presented in Table 5, which shows no significant differences on any individual MISL dimension (all p > .05). Although the composite Knowledge Construction measure (KCO_avg) approached significance (p = 0.065) in the univariate analysis, this effect did not reach the conventional threshold. Other variables also failed to show significant differences (AIR, p = 0.239; SAM, p = 0.258; PAC, p = 0.685; ASF, p = 0.098). These findings suggest that prior flipped learning experience did not confer an advantage in students’ perception of their learning experience within the current instructional design. Both groups demonstrated consistently high satisfaction across all dimensions, with mean scores ranging from 4.27 to 4.58, indicating that implementation quality and design factors may be more influential than prior familiarity in shaping student perceptions of flipped classroom effectiveness.

**Table 5:** Univariate ANOVA results for each MISL dimension.

MISL Dimension	F	df	p-value	Partial η²	No Prior Experience M (SD)	Prior Experience M (SD)
AIR	1.395	1,295	0.239	.005	4.48 (0.66)	4.40 (0.86)
SAM	1.283	1,295	0.258	.004	4.54 (0.60)	4.46 (0.83)
PAC	0.165	1,295	0.685	.001	4.31 (0.87)	4.27 (0.91)
ASF	2.763	1,295	0.098	.009	4.47 (0.60)	4.36 (0.80)
KCO	3.422	1,295	0.065	0.011	4.58 (0.62)	4.47 (0.82)

### 4.4 Qualitative insights from open-ended responses

To complement and contextualize the quantitative findings, students answered three open-ended questions at the end of the questionnaire about their experience with the flipped classroom. A thematic analysis was conducted, identifying recurring patterns in responses. Below is a synthesis of the key themes. The links between these themes and the survey data are explored in the integration subsection that follows.

**Q1. How did preparing at home change the way you participate in class?**

Table 6 summarizes the themes and illustrative examples from responses to Q1. Students overwhelmingly reported that preparing at home enhanced their participation and confidence during in-class sessions. Key sub-themes included:

**Table 6:** Themes and illustrative examples from responses to Q1: “How did preparing at home change the way you participate in class?”.

Themes	Examples
Greater preparedness and understanding: Many students stated they arrived to class “ready,” which made it easier to follow along, understand concepts, and answer questions.	“When I prepare at home, I understand more in class and I can speak more.”
Increased participation and confidence: Several participants mentioned feeling more confident speaking up and engaging in discussions.	“Preparing at home increased my confidence and participation in class.”
Improved time use in class: Some students appreciated that the class could focus more on practice and application rather than explanation.	“It saved class time for interaction.”
Responsibility and autonomy: Preparing before class made students feel more responsible for their own learning, echoing a core constructivist principle.	“It made me more responsible for my learning.”

**Q2. What stands out most about your experience with the flipped classroom?**

Responses revealed highly positive sentiments about the interactive, engaging, and student-centered nature of the flipped classroom. As shown in Table 7, prominent themes that emerged from Q2 responses included:

**Table 7:** Themes and illustrative examples from responses to Q2: “What stands out most about your experience with the flipped classroom?”.

Themes	Examples
Increased engagement and interactivity: Students emphasized how the flipped model shifted learning from passive to active.	“It was more interactive and fun, not just listening to the teacher.”
Usefulness and motivation: Learners found the structure motivational and well-organized, helping them stay focused.	“It made me more motivated and helped me practice more.”
Collaborative learning: Students appreciated opportunities to work with peers.	“Group work and classroom interaction helped me speak more.”
Supportive teaching approach: Multiple students praised instructors for their explanations, patience, and use of examples.	“The teacher’s way of explaining made it easier.”

**Q3. Additional comments about the course**

The majority of additional comments were positive, highlighting overall satisfaction, but several constructive suggestions also emerged. Table 8 outlines the themes and representative comments from Q3, where students shared additional reflections:

**Table 8:** Themes and illustrative examples from responses to Q3: “Additional comments about the course”.

Themes	Examples
Positive overall experience: Many responses expressed deep appreciation for the course and the flipped approach.	“This was a great course. I loved learning in this way.”
Desire for continued access to resources: A recurring theme was the request to keep Moodle open after course completion for revision and ongoing learning.	“Please let us keep access to Moodle after the course ends.”
Requests for additional practice: Students requested more speaking, grammar, and pronunciation exercises, both in class and online.	“We need more speaking activities and grammar tasks.”
Suggestions for improvement: Some students suggested improvements in Moodle usability, the addition of downloadable materials, and greater flexibility in course booking.	“Sometimes Moodle is hard to access. We need downloadable PDFs.”

The open-ended responses support the quantitative findings by providing nuanced insight into how and why the flipped model was effective. Consistent with Awidi’s [17,18] constructivist model, students reported that access to materials, motivation, participation, and feedback were all enhanced by the flipped design. Critically, students also offered valuable recommendations for sustaining and improving the flipped learning experience, particularly in terms of platform access and resource availability.

### 4.5 Sentiment analysis

To complement the quantitative findings, sentiment analysis was conducted on responses to the three open-ended survey questions/statements. For clarity and consistency, results for each question are reported under the following labels across text, descriptive statistics, and all figures:

Q1: “How did preparing at home change the way you participate in class?” — Participation Change

Q2: “What stands out to you the most about your experience with the flipped classroom approach in this course?” — Flipped Experience

Q3: “Please add any comments about your experience in this course.” — Open Comments

Using TextBlob, each response was evaluated for sentiment on a polarity scale ranging from −1 (highly negative) to +1 (highly positive). The resulting sentiment scores provided insight into students’ affective responses toward the flipped classroom experience.

The mean sentiment scores for each question were as follows: Participation Change (Q1): 0.120972, Flipped Experience (Q2): 0.115227, and Open Comments (Q3): 0.185250. These results suggest a general trend of positive sentiment, with the Open Comments category reflecting the most favorable attitudes. This may indicate that when given freedom to articulate their thoughts without prompt structure, students tended to express satisfaction and appreciation for the approach.

**Fig 1:**
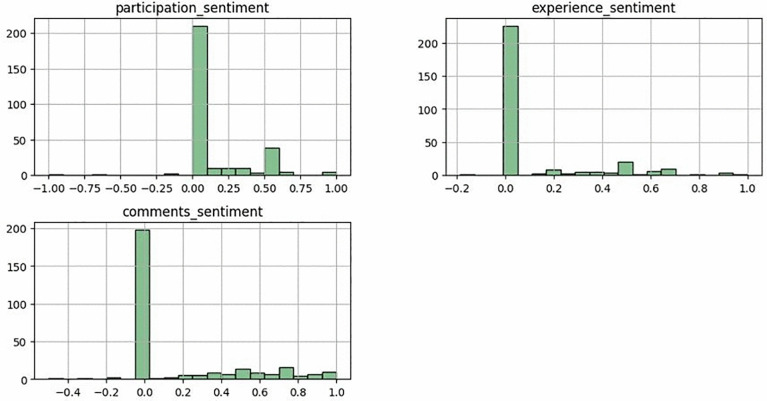
Histograms of sentiment polarity scores by category: Participation Change (Q1), Flipped Experience (Q2), and Open Comments (Q3).

**Fig 2:**
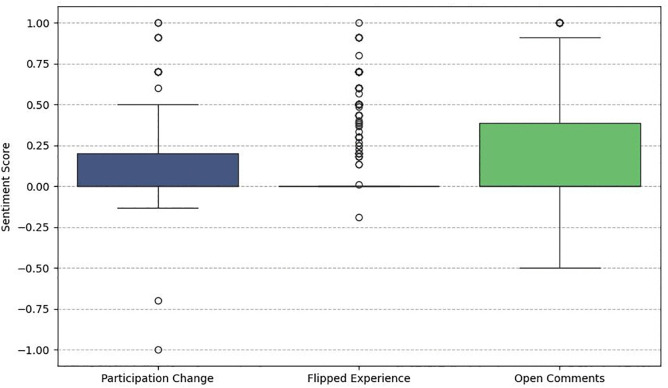
Boxplots of sentiment polarity scores by category: Participation Change (Q1), Flipped Experience (Q2), and Open Comments (Q3).

**Fig 3:**
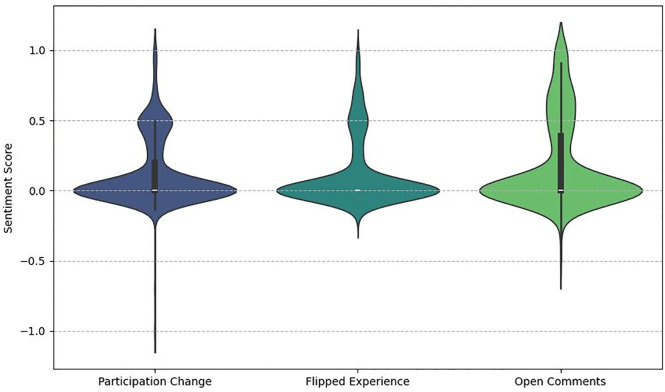
Violin plots showing the distribution of sentiment polarity scores by category: Participation Change (Q1), Flipped Experience (Q2), and Open Comments (Q3).

To explore the distribution and variability of sentiment, visualizations including histograms (Fig 1), boxplots (Fig 2), and violin plots (Fig 3) were employed. While the average sentiment across all three categories remained positive, the distribution plots revealed notable variation, particularly in the Participation Change responses, which exhibited a wider spread and a higher frequency of neutral or slightly negative sentiments.

The boxplots (Fig 2) provided a clear depiction of the median sentiment values and interquartile ranges, suggesting that while the central tendency leaned positive, there was non-negligible variability. The violin plots (Fig 3), moreover, illustrated density and multimodal tendencies, offering deeper insight into where student sentiment clustered, most commonly around the slightly positive range.

These findings underscore a generally positive student reception of the flipped classroom experience, while also highlighting the presence of diverse individual experiences. The variation in sentiment, particularly observed in the participation change responses, suggests that the impact of preparing at home on class participation may be perceived differently by students. Future research could study in-depth the qualitative data to identify specific factors contributing to both positive and negative sentiment within each category, providing more understanding of the student experience.

## 5. Discussion

The present study offers robust, triangulated evidence from quantitative, qualitative, and sentiment analyses supporting the effectiveness of the flipped classroom model in general English instruction, as perceived by adult learners in an Egyptian continuing education context. The flipped classroom approach, which reorganizes instruction to promote active, self-directed learning and enhanced in-class engagement, has been widely recognized as an effective pedagogical strategy in EFL contexts [15,31], providing an ideal framework for examining how pedagogical innovation influences the learning experience. Drawing on Awidi’s [17,18] Model for Improving Student Learning (MISL), the findings illustrate how flipped pedagogy can positively influence multiple dimensions of the learning experience, including access to resources, learner motivation, participation, assessment practices, and perceived understanding. These outcomes align with constructivist learning theory’s emphasis on active engagement and personal meaning-making in the learning process [19].

### 5.1 The pedagogical value of flipped learning

Quantitative results revealed consistently high mean scores across all five MISL constructs, suggesting that students recognized substantial value in the flipped classroom approach. Most notably, Knowledge Construction (KCO) emerged as the strongest dimension, indicating that students felt they deeply internalized what they learned through this pedagogical model. The means of Support and Motivation (SAM) and Assessment and Feedback (ASF) followed KCO closely, indicating that students recognized tangible benefits from engaging with materials prior to class. These positive outcomes align with the pedagogical foundations established by Bergmann and Sams [6] and Bishop and Verleger [7], who emphasize active, self-directed learning and higher-order cognitive engagement in flipped environments. The findings are further supported by extensive research documenting flipped classroom efficacy in EFL contexts, particularly Egyptian studies reporting gains in language skills, motivation, and learner autonomy [11,13,14,30]. Broader meta-analytic evidence by Shao and Liu [8] confirms increased engagement and achievement across disciplines while acknowledging that outcomes depend on implementation quality and contextual factors.

For ASF, however, item-level examination showed important variations; while instant feedback received overwhelming endorsement, assessment format preferences were more divided, with a notable minority preferring traditional in-class quizzes over online practice questions. Participation in Learning Activities (PAC) revealed the greatest complexity, with frequency data showing that while preparation-related benefits were widely recognized, competitive elements such as Moodle leaderboards generated more mixed responses. This variability in student preferences reflects the heterogeneous nature of adult continuing education populations, where learners bring diverse comfort levels with collaborative learning, competition, and public participation, elements that traditional classroom designs may not fully accommodate. These patterns align with previous research documenting variability in flipped learning engagement and the influence of cultural and contextual factors on student outcomes [4,8,31].

Furthermore, regression analysis provided key insights into the main drivers of student learning success. The analysis revealed that learner confidence, personal motivation, and engagement were strong predictors of perceived learning outcomes, while digital competence was a comparatively weaker predictor. This indicates that students’ attitudes and beliefs about learning have greater influence than technical skills in adapting to flipped environments. Notably, learner confidence was the most significant predictor, underscoring its vital role in adult education, where self-efficacy drives motivation, engagement, and persistence. This aligns with Bandura’s [23] self-efficacy theory and is supported by findings from Lestari [32], emphasizing the importance of confidence and motivation among adult learners, as well as Knowles’ [22] andragogical principles. These results reaffirm that self-regulation and belief in one’s abilities are essential, especially in innovative models like flipped classrooms where learners face new roles and challenges. For adults balancing multiple commitments, confidence mediates willingness to tackle challenges and persistence through early difficulties. Additionally, the secondary role of digital competence challenges the assumption that technical proficiency is a major barrier to flipped classroom success. Instead, adult EFL learners effectively engage with technology-enhanced learning when provided with appropriate pedagogical scaffolding and affective support. This concurs with Chang [10] and Yilmaz [16], who found that learner readiness factors outweigh technical skills.

Interestingly, the MANOVA results indicated that prior exposure to flipped classrooms did not significantly influence students’ perceptions of their current learning experience. It had been hypothesized that such prior exposure would positively impact students’ learning outcomes. Given its categorical nature (Yes/No), the variable was analyzed separately. However, the findings challenge the assumption that familiarity with the flipped model automatically enhances adaptation or satisfaction. Instead, the results suggest that well-designed flipped instruction, regardless of students’ prior experience, can be equally effective for both novice and experienced learners when appropriate scaffolding is in place. This echoes Lo and Hew [20] and Lestari [32], who argued that implementation quality and learner support are more influential than familiarity alone in shaping perceptions and success.

### 5.2 Integrating statistical, thematic, and sentiment analyses

Thematic analysis of open-ended responses provided depth and contextual richness to the statistical results. Students consistently reported that preparing at home improved their readiness, increased their confidence, and allowed for more interactive and student-centered classroom experiences. These insights align closely with the quantitative findings on participation and engagement, reinforcing the value of pre-class preparation in fostering active learning. Students particularly emphasized how preparation enabled them to take greater responsibility for their learning and engage more meaningfully in collaborative activities, echoing findings from Egyptian flipped classroom studies that highlight enhanced learner autonomy and peer collaboration [11,13,30]. However, requests for more speaking and grammar practice and extended resource access on Moodle illustrate that even within positive dimensions, opportunities for refinement remain. These qualitative insights underscore the critical importance of self-directed learning capabilities and learner autonomy in successful flipped learning environments, aligning with broader flipped EFL research [4,10]. The challenges are particularly pronounced among adult students, who must balance course preparation with work and family commitments. The requests for extended access and more practice opportunities reflect the unique needs of continuing education learners who may benefit from extended engagement periods beyond traditional semester boundaries.

Affective dimensions of the learning experience were further illuminated through sentiment analysis. Although average sentiment scores across the three open-ended questions were modestly positive, the open comments section yielded the highest polarity score, suggesting that when students were free to express themselves without a specific prompt, their reflections were more overtly appreciative and affirming. Nevertheless, the wider sentiment distribution in responses about participation change mirrors the quantitative variability observed in the PAC dimension, providing convergent evidence of variability in how students experienced pre-class preparation. For some, it increased autonomy and confidence; for others, it may have introduced new challenges or reinforced existing anxieties, consistent with meta-analytic findings documenting variability in flipped classroom outcomes [8].

The integrated summary in Table 9 brings these multiple data strands together, showing substantial agreement between mean scores, frequency data, thematic trends, and sentiment patterns. Table 9 particularly highlights how PAC’s variability appears consistently across all data types, while KCO shows the strongest alignment across all measures, reinforcing its position as the most successful aspect of the flipped classroom implementation. Taken together, the quantitative, qualitative, and sentiment findings confirm that while the flipped model was largely well-received, student experiences were not monolithic. This highlights the importance of differentiated support and inclusive design, particularly for learners who may struggle with independent preparation or lack access to reliable digital infrastructure.

**Table 9:** Integration of quantitative, qualitative, and sentiment findings across MISL dimensions.

MISL dimension	Quantitative results (M, % Agreement)	Item‑Level High/Low	Key qualitative themes	Sentiment analysis (Mean Polarity; Distribution Notes)	Nature of convergence
AIR	M = 4.44 (SD = 0.79); 86.7% agree	**High:** 88.2% agreed Moodle materials helped them understand topics and outcomes. **Low:** 85.9% agreed they had necessary resources to complete the course.	Stronger preparedness for class; easy access to materials; requests for post‑course Moodle access	0.120972; positive, narrow range	Strong convergence; small access issues noted despite overall satisfaction
SAM	M = 4.49 (SD = 0.73); 87.9% agree	**High:** 89.6% enjoyed interactive activities (and same % found them easy to use). **Low:** 84.5% felt preparing at home was better than traditional learning.	Increased motivation; greater responsibility for learning; enjoyment of interactive tasks	0.115227; consistently positive	Strong convergence; sentiment reflects high engagement
PAC	M = 4.30 (SD = 0.90); 80.2% agree	**High:** 85.9% said preparation improved participation. **Low:** 77.4% valued leaderboard competition (same % said groups were prepared).	More active participation; enhanced collaboration; mixed reception of competitive elements; platform usability suggestions	0.120972; widest sentiment range	Mostly convergent; both sentiment and frequencies show variability
ASF	M = 4.42 (SD = 0.70); 85.2% agree	**High:** 91.9% valued instant feedback. **Low:** 70.7% preferred online to in‑class quizzes.	Valued clear, timely feedback; diverse assessment preferences	0.115227; overall positive with few negatives	Convergent; high agreement but format preference diversity
KCO	M = 4.52 (SD = 0.73); 87.5% agree	**High:** 88.2% valued in‑class speaking/writing tasks. **Low:** 87.2% felt online assessments improved course understanding (same % for Moodle prep improving English).	Deeper understandin; strong retention; requests for more practice	0.185250; most positive polarity	Strongest convergence; sentiment and themes reinforce top rating

Note: Means (M), standard deviations (SD), and frequency percentages are from Likert‐type items. Sentiment polarity ranges from −1 = very negative to +1 = very positive.

### 5.3 Implications for continuing education program design

This study affirms the validity of flipped learning in continuing education environments, where adults balance multiple responsibilities while pursuing diverse learning goals. These findings emphasize the critical importance of tailoring flipped pedagogies to adult learners’ unique needs to support both retention and satisfaction. At a programmatic level, for continuing education programs serving adult learners similar to our sample, these findings emphasize several key considerations. First, successful flipped classroom implementation requires deliberate decision-making regarding technology selection and thoughtful evaluation of existing institutional infrastructure alongside comprehensive instructor professional development. Teachers need training not only in flipped pedagogical principles but also in digital facilitation skills that accommodate varying technological proficiencies among adult students. These implementation challenges mirror those documented by Kakarougkas and Abdellatif [24], who emphasize the importance of comprehensive support systems in Middle Eastern educational contexts. Second, the study’s findings have important implications for resource allocation, suggesting that institutions should prioritize user-friendly platforms and ongoing technical support over assuming high baseline digital competency. Finally, the scalability of flipped approaches across different English proficiency levels warrants attention in continuing education settings where students often enter with varied linguistic backgrounds and learning goals. While the flipped classroom demonstrates considerable promise for adult continuing education, sustainability and learner diversity issues require ongoing attention. Recent comprehensive reviews underscore the necessity of addressing long-term effectiveness and demographic variations within educational contexts [4,8].

## 6. Conclusion

This study provides compelling evidence for the pedagogical value of the flipped classroom model for adult learners in general English instruction within continuing education contexts and programs. Drawing on a mixed-methods design and guided by Awidi’s [17,18] constructivist framework, the research revealed that students perceived substantial benefits in terms of motivation, engagement, access to resources, and perceived learning outcomes. Regression analyses confirmed that affective and metacognitive factors, particularly learner confidence, were significant predictors of perceived learning, while digital competence, though significant, was a less influential factor. This highlights the centrality of learner mindset, self‑belief, and self‑regulation in technology‑enhanced environments, aligning with previous socio‑constructivist literature.

Thematic and sentiment analyses enriched these findings with nuanced insights into students’ lived experiences, revealing both strong positive perceptions and important variability in how learners experienced the flipped format. While the majority embraced the autonomy and interactivity of flipped learning, others showed mixed responses to competitive classroom elements and assessment formats, requesting improved platform accessibility and downloadable materials. Importantly, this variability should not be viewed as a limitation but rather as valuable guidance for designing adaptable, differentiated learning environments. The findings reinforce that implementation quality and embedded learner supports, rather than prior familiarity with flipped learning or advanced digital skills, most strongly influence student perceptions and success.

It is important to note that these findings represent the experiences of adult learners enrolled in a general English course at a continuing education institution in Egypt. While the methodology and insights may inform practice in similar contexts, broader generalization to other proficiency levels or geographic regions should be pursued cautiously and in concert with local contextual evaluation.

### 6.1 Recommendations for practice

Based on the findings, several recommendations can be made for practitioners, researchers, and institutions implementing flipped learning. Design for diversity: Recognizing that students vary in their readiness for independent learning, flipped instruction should include scaffolds such as pre-class guidance, flexible timelines, and varied media formats to support different learning styles and competencies. Enhance platform accessibility: Given students’ repeated requests for ongoing access to Moodle and downloadable content, institutions should consider extending platform availability post-course and ensuring that learning materials are accessible offline where possible. Increase in-class interactivity: The flipped model thrives when class time is used for active learning. More speaking tasks, collaborative exercises, and formative feedback activities should be embedded to maximize engagement. Monitor affective responses: Since learner confidence strongly predicts perceived learning, teachers should monitor and support affective dimensions such as anxiety, motivation, and self-efficacy, particularly in the early stages of flipped implementation. Incorporate student feedback: Continuous feedback loops should be institutionalized to allow iterative improvements based on student suggestions, especially related to usability, flexibility, and content balance.

### 6.2 Limitations and future research

While this study provides valuable insights, several limitations should be acknowledged: Context-specificity: The study was conducted within a single institutional and disciplinary context (general English education), which may limit the generalizability of findings to other disciplines or educational levels. Self-report bias: The data relied primarily on self-reported measures, which may be subject to social desirability or recall biases. Sentiment tool limitations: While TextBlob provided a useful overview of affective responses, it lacks the linguistic sophistication to capture complex emotions in non-English texts. Future studies could explore more advanced sentiment analysis models or apply human-coded sentiment validation. Limited longitudinal scope: The research captured perceptions at a single time point. Longitudinal studies would be beneficial in assessing how attitudes and outcomes evolve over time, particularly as students adapt to the flipped format.

Future research should continue to explore how flipped models can be optimized across disciplines and learner profiles, ensuring inclusive and sustainable innovation in continuing education and lifelong learning contexts.

### 6.3 Overall contribution

This study presents an innovative integration of mixed-methods research with computational sentiment analysis, establishing a comprehensive analytical framework that captures both quantitative patterns and nuanced affective insights in educational evaluation. The research addresses a critical gap in adult continuing education, an area with profound implications for pedagogy yet severely underrepresented in flipped classroom literature. The findings reinforce the potential of the flipped classroom as a student-centered pedagogical approach while demonstrating that implementation quality and learner support outweigh technological sophistication. Moreover, learner variation emerges not as a challenge but as valuable guidance for designing adaptable, inclusive learning environments. These insights hold particular significance for resource-constrained and non-Western contexts, where pedagogical innovation must transcend infrastructure limitations.
