# Supplementary material for: Effectiveness of flipped classroom pedagogy: A mixed-methods analysis of adult continuing education—Case study, Egyptian context
Source: PLoS One. 2026 Jul 16;21(7):e0353699. doi: 10.1371/journal.pone.0353699 (PMC13375013; doi:10.1371/journal.pone.0353699)
Supplement: Attachment — Submitted filename: Response to Reviewers.docx [file pone.0353699.s003.docx]

Dear Editor,

Thank you for your final review and for your helpful comments on our manuscript, [PONE-D-25-50019], “Effectiveness of Flipped Classroom Pedagogy: A Mixed-Methods Analysis of Adult Continuing Education—Case Study, Egyptian Context.” We appreciate your time and guidance in helping us refine our paper for publication.

Below, we provide a point-by-point response to each of your comments, along with the actions taken to address them.

| **Editor’s Comment** | **Authors’ Response** | **Changes Made** |
| --- | --- | --- |
| 1. Please number the research questions, rather than presenting them as multiple paragraphs. | Accepted and addressed. | All research questions have been clearly numbered for better readability and structural clarity. |
| 2. Please use complete sentences when referring to the literature. For example, “ conducted a controlled university study…” should read as “Author(s)’ names conducted…” The authors should check the entire paper to correct this issue consistently. | Accepted and addressed. | We have thoroughly reviewed the entire manuscript to ensure that all literature references are now presented in complete sentences incorporating author names before the citations. This correction has been applied consistently throughout the entire paper. |

We trust that these revisions fully address your remaining concerns. We are grateful for your time and consideration, and we look forward to your final decision regarding the acceptance of our manuscript.

Kind regards,
The authors
