# Supplementary material for: Effectiveness of flipped classroom pedagogy: A mixed-methods analysis of adult continuing education—Case study, Egyptian context
Source: PLoS One. 2026 Jul 16;21(7):e0353699. doi: 10.1371/journal.pone.0353699 (PMC13375013; doi:10.1371/journal.pone.0353699)
Supplement: Attachment — Submitted filename: Response to Reviewers for Manuscript [PONE-D-25-50019].docx [file pone.0353699.s002.docx]

Dear Editor and Reviewers,

Thank you for your thoughtful review and constructive comments on our manuscript, [PONE-D-25-50019], “Effectiveness of Flipped Classroom Pedagogy: A Mixed-Methods Analysis of Adult Continuing Education in a Middle Eastern Context.” We appreciate your time and valuable feedback, which has helped us improve the quality of our work. Below, we provide a point-by-point response to each comment, along with details of the changes made.

**All reviewers’ comments were accepted, and changes to the manuscript were made accordingly.**

| Points raised by Reviewer 1, Dr. Mohd Elmagzoub Eltahir | | |
| --- | --- | --- |
| Reviewer Comment | Authors’ Response | Changes Made |
| Explicitly link Awidi’s (2006a, 2006b) Model for Improving Student Learning (MISL) to adult-learning and self-efficacy frameworks (e.g., Knowles’ andragogy; Bandura, 1997). | Accepted and Addressed | A new theoretical synthesis paragraph has been added in Section 2.2 (Literature Review) linking Awidi's MISL dimensions to Knowles (1974, 1980) and Bandura (1997), with specific examples of how AIR, SAM, PAC, ASF, and KCO correspond to adult learning principles and self-efficacy constructs. |
| Merge overlapping definitions and repeated citations in Sections 2.1–2.3 (e.g., Bergmann & Sams 2012; Bishop & Verleger 2013; Shao & Liu 2021; Pongpanich et al., 2025) into one concise overview. | Accepted and Addressed | Sections 2.1–2.3 have been reviewed to streamline and eliminate overlaps in definitions and citations. |
| Add a Data Availability Statement specifying where anonymized datasets, coding frameworks, or sentiment-analysis scripts can be accessed. | Accepted and Addressed | A Data Availability Statement has been prepared specifying that anonymized questionnaire datasets, qualitative coding framework, and Python-based sentiment analysis scripts are available upon request from researchers. Due to ethical restrictions protecting participant privacy (IRB approval#2024-2025-278), the statement clarifies procedures for researchers to contact the corresponding author (H. Said, halaalaa@aucegypt.edu) for data access. This statement is submitted in the "Additional Information" section during revision submission. |
| Points raised by Reviewer 2, Dr. Abdellatef Alqawasmi | | |
| Revise the manuscript title to better reflect its scope and context, for example:  “Effectiveness of Flipped Classroom Pedagogy: A Mixed-Methods Analysis of Adult Continuing Education—Case Study, Egypt Context.” | Accepted and Addressed | The manuscript title was revised to 'Effectiveness of Flipped Classroom Pedagogy: A Mixed-Methods Analysis of Adult Continuing Education—Case Study, Egyptian Context.' |
| Refine the research questions to explicitly highlight the adult continuing-education population. For example:  “How do adult students in a continuing education program perceive the flipped classroom experience in general English instruction across the five dimensions of Awidi’s constructivist framework?” | Accepted and Addressed | All research questions have been revised to explicitly reference the adult continuing-education student population, clearly reflecting the study's scope and context. |
| Rewrite the discussion and recommendations to avoid overgeneralization and ensure conclusions align closely with the sample’s boundaries. | Accepted and Addressed | We have comprehensively revised the Discussion and Conclusion sections to ensure precise alignment with our sample's specific characteristics and boundaries. The revisions include: (1) explicitly identifying our population as adult learners in a continuing education context in the opening paragraph; (2) qualifying all findings and implications with references to the specific sample characteristics (proficiency level A1D, single institution, Egyptian context); (3) adding a prominent scope acknowledgment in the Conclusion stating that findings represent this specific population and should not be generalized to other proficiency levels or regions without additional research; and (4) expanding the Limitations section to further emphasize context-specificity. These revisions ensure readers understand precisely which population and contexts the findings apply to, while maintaining the evidential strength of our mixed-methods analyses. |

We trust that the revisions and clarifications provided adequately address the suggestions made by the reviewers. We look forward to receiving the final acceptance on our manuscript and getting the paper published.

Kind regards,

The authors
