# Supplementary material for: Effectiveness of flipped classroom pedagogy: A mixed-methods analysis of adult continuing education—Case study, Egyptian context
Source: PLoS One. 2026 Jul 16;21(7):e0353699. doi: 10.1371/journal.pone.0353699 (PMC13375013; doi:10.1371/journal.pone.0353699)
Supplement: Attachment — Submitted filename: Response to Reviewers for Manuscript.docx [file pone.0353699.s004.docx]

Dear Editor and Reviewer,

Thank you for your final review and for your helpful comments on our manuscript, [PONE-D-25-50019], “Effectiveness of Flipped Classroom Pedagogy: A Mixed-Methods Analysis of Adult Continuing Education—Case Study, Egyptian Context.”

We are grateful to Reviewer #3 for confirming that all prior concerns have been addressed. The reviewer's positive evaluation of the study's methodological rigor, statistical analyses, theoretical framework, and written presentation is deeply encouraging and reflects the substantial effort invested across all revision rounds. The sole outstanding issue, data accessibility, has now been fully resolved, as detailed below.

| **Comment** | **Authors’ Response** | **Changes Made** |
| --- | --- | --- |
| PLOS ONE requires publicly accessible underlying data. The authors should confirm whether a fully anonymized dataset can be deposited in a public repository. | Accepted and addressed. | A fully anonymized dataset has been deposited in the Zenodo public repository (https://doi.org/10.5281/zenodo.20363249), with all identifying variables removed. The Data Availability Statement has been updated accordingly. |

We are confident this revision fully addresses all outstanding concerns and that the manuscript now meets PLOS ONE's publication requirements. We sincerely thank the Academic Editor and the Reviewers for their time, constructive guidance, and encouragement throughout this process, and we look forward to a favorable final decision.

Kind regards,
The authors
