# Supplementary material for: Effectiveness of flipped classroom pedagogy: A mixed-methods analysis of adult continuing education—Case study, Egyptian context
Source: PLoS One. 2026 Jul 16;21(7):e0353699. doi: 10.1371/journal.pone.0353699 (PMC13375013; doi:10.1371/journal.pone.0353699)
Supplement: Peer review history

# Peer review: Decision Letter 0

**DOI:** 10.1371/journal.pone.0353699.r001

**Authors:** Najeh Alsalhi

3 Nov 2025

PONE-D-25-50019

Effectiveness of Flipped Classroom Pedagogy: A Mixed-Methods Analysis of Adult Continuing Education in a Middle Eastern Context

PLOS ONE

Dear Dr. Said,

Thank you for submitting your manuscript to PLOS ONE. After careful consideration, we feel that it has merit but does not fully meet PLOS ONE’s publication criteria as it currently stands. Therefore, we invite you to submit a revised version of the manuscript that addresses the points raised during the review process.

Please submit your revised manuscript by Dec 18 2025 11:59PM. If you will need more time than this to complete your revisions, please reply to this message or contact the journal office at plosone@plos.org. When you're ready to submit your revision, log on to https://www.editorialmanager.com/pone/ and select the ’submissions Needing Revision' folder to locate your manuscript file.

Please include the following items when submitting your revised manuscript:

- A rebuttal letter that responds to each point raised by the academic editor and reviewer(s). You should upload this letter as a separate file labeled 'Response to Reviewers'.
- A marked-up copy of your manuscript that highlights changes made to the original version. You should upload this as a separate file labeled 'Revised Manuscript with Track Changes'.
- An unmarked version of your revised paper without tracked changes. You should upload this as a separate file labeled 'Manuscript'.

-->If you would like to make changes to your financial disclosure, please include your updated statement in your cover letter. Guidelines for resubmitting your figure files are available below the reviewer comments at the end of this letter.

If applicable, we recommend that you deposit your laboratory protocols in protocols.io to enhance the reproducibility of your results. Protocols.io assigns your protocol its own identifier (DOI) so that it can be cited independently in the future. For instructions see: https://journals.plos.org/plosone/s/submission-guidelines#loc-laboratory-protocols. Additionally, PLOS ONE offers an option for publishing peer-reviewed Lab Protocol articles, which describe protocols hosted on protocols.io. Read more information on sharing protocols at https://plos.org/protocols?utm_medium=editorial-email&utm_source=authorletters&utm_campaign=protocols.

We look forward to receiving your revised manuscript.

Kind regards,

Najeh Rajeh Ibrahim Alsalhi, Ph.D.

Academic Editor

PLOS ONE

Journal requirements:

When submitting your revision, we need you to address these additional requirements.

1. Please ensure that your manuscript meets PLOS ONE’s style requirements, including those for file naming. The PLOS ONE style templates can be found at

https://journals.plos.org/plosone/s/file?id=wjVg/PLOSOne_formatting_sample_main_body.pdf and

https://journals.plos.org/plosone/s/file?id=ba62/PLOSOne_formatting_sample_title_authors_affiliations.pdf

2. Your ethics statement should only appear in the Methods section of your manuscript. If your ethics statement is written in any section besides the Methods, please move it to the Methods section and delete it from any other section. Please ensure that your ethics statement is included in your manuscript, as the ethics statement entered into the online submission form will not be published alongside your manuscript.

3. Please include a separate caption for each figure in your manuscript.

4. If the reviewer comments include a recommendation to cite specific previously published works, please review and evaluate these publications to determine whether they are relevant and should be cited. There is no requirement to cite these works unless the editor has indicated otherwise.

5. Please review your reference list to ensure that it is complete and correct. If you have cited papers that have been retracted, please include the rationale for doing so in the manuscript text, or remove these references and replace them with relevant current references. Any changes to the reference list should be mentioned in the rebuttal letter that accompanies your revised manuscript. If you need to cite a retracted article, indicate the article’s retracted status in the References list and also include a citation and full reference for the retraction notice.

Additional Editor Comments:

Dear Ms. Hala Mohamed Said,

Thank you for submitting your manuscript titled “Effectiveness of Flipped Classroom Pedagogy: A Mixed-Methods Analysis of Adult Continuing Education in a Middle Eastern Context” to PLOS ONE.

The reviewers have completed their evaluations. Both recognize the scholarly value of your study, and they commend its methodological rigor and contribution to the literature on adult education and flipped learning. However, they have suggested several minor revisions to improve clarity, precision, and focus before final acceptance.

Please address the following points in your revised manuscript:

Reviewer 1 (Minor Revision):

Explicitly link Awidi’s (2006a, 2006b) Model for Improving Student Learning (MISL) to adult-learning and self-efficacy frameworks (e.g., Knowles’ andragogy; Bandura, 1997).

Merge overlapping definitions and repeated citations in Sections 2.1–2.3 (e.g., Bergmann & Sams 2012; Bishop & Verleger 2013; Shao & Liu 2021; Pongpanich et al., 2025) into one concise overview.

Add a Data Availability Statement specifying where anonymized datasets, coding frameworks, or sentiment-analysis scripts can be accessed.

Reviewer 2 (Accept):

Revise the manuscript title to better reflect its scope and context, for example:

“Effectiveness of Flipped Classroom Pedagogy: A Mixed-Methods Analysis of Adult Continuing Education—Case Study, Egypt Context.”

Refine the research questions to explicitly highlight the adult continuing-education population. For example:

“How do adult students in a continuing education program perceive the flipped classroom experience in general English instruction across the five dimensions of Awidi’s constructivist framework?”

Rewrite the discussion and recommendations to avoid overgeneralization and ensure conclusions align closely with the sample’s boundaries.

We look forward to receiving your revised manuscript within 14 days of this decision letter.

Thank you for your valuable contribution to the field.

Sincerely,

Dr. Najeh Rajeh Ibrahim Alsalhi

Academic Editor, PLOS ONE

[Note: HTML markup is below. Please do not edit.]

Reviewers' comments:

Reviewer’s Responses to Questions

**Comments to the Author**

1. Is the manuscript technically sound, and do the data support the conclusions?

The manuscript must describe a technically sound piece of scientific research with data that supports the conclusions. Experiments must have been conducted rigorously, with appropriate controls, replication, and sample sizes. The conclusions must be drawn appropriately based on the data presented.

Reviewer #1: Yes

Reviewer #2: Yes

**********

2. Has the statistical analysis been performed appropriately and rigorously?

Reviewer #1: Yes

Reviewer #2: Yes

**********

3. Have the authors made all data underlying the findings in their manuscript fully available?

The PLOS Data policy requires authors to make all data underlying the findings described in their manuscript fully available without restriction, with rare exception (please refer to the Data Availability Statement in the manuscript PDF file). The data should be provided as part of the manuscript or its supporting information, or deposited to a public repository. For example, in addition to summary statistics, the data points behind means, medians and variance measures should be available. If there are restrictions on publicly sharing data—e.g. participant privacy or use of data from a third party—those must be specified.

Reviewer #1: No

Reviewer #2: Yes

**********

4. Is the manuscript presented in an intelligible fashion and written in standard English?

PLOS ONE does not copyedit accepted manuscripts, so the language in submitted articles must be clear, correct, and unambiguous. Any typographical or grammatical errors should be corrected at revision, so please note any specific errors here.

Reviewer #1: Yes

Reviewer #2: Yes

**********

5. Review Comments to the Author

Please use the space provided to explain your answers to the questions above. You may also include additional comments for the author, including concerns about dual publication, research ethics, or publication ethics. (Please upload your review as an attachment if it exceeds 20,000 characters)

Reviewer #1: This manuscript presents a rigorous and well-executed mixed-methods investigation into the effectiveness of a flipped-classroom model within an adult EFL continuing-education context in Egypt. It offers an original contribution by triangulating quantitative, qualitative, and computational sentiment analyses an approach that meaningfully extends conventional evaluation methods in technology-enhanced learning. The study is technically sound, ethically compliant, and clearly written. The conclusions are proportionate to the data and underscore the pedagogical and affective dimensions of flipped learning in non-Western contexts.

That said, several areas would benefit from focused revision to strengthen clarity, concision, and transparency:

1- Explicitly link Awidi’s (2006a, 2006b) Model for Improving Student Learning (MISL) to adult-learning and self-efficacy frameworks (e.g., Knowles’ andragogy; Bandura, 1997). A short mapping paragraph will clarify how MISL dimensions correspond to affective and metacognitive constructs such as confidence and motivation.

2- Sections 2.1–2.3 contain overlapping definitions and repeated citations (e.g., Bergmann & Sams 2012; Bishop & Verleger 2013; Shao & Liu 2021; Pongpanich et al. 2025). Merge these into one concise overview.

3- Add a Data Availability Statement specifying where anonymized datasets, coding frameworks, or sentiment-analysis scripts can be accessed.

Reviewer #2: The manuscript addresses a highly important topic—specifically, the teaching method of flipped learning and the study population, which consists of adult students in a continuing education program, as well as the research questions it seeks to answer.

The manuscript presents an acceptable systematic review and highlights a clear research gap: most existing studies still focus primarily on younger or full-time students, with limited attention given to adult learners or those engaged in continuing education.

Regarding the title, “Effectiveness of Flipped Classroom Pedagogy: A Mixed-Methods Analysis of Adult Continuing Education in a Middle Eastern Context”, it is overly broad. I do not share the authors’ view, as the manuscript is essentially a case study in Egypt—specifically, at a distinctive institution that does not fully represent Egyptian society: the American University in Cairo (AUC). Therefore, the results cannot be generalized to the entire Middle East region. I recommend revising the title as follows:

“Effectiveness of Flipped Classroom Pedagogy: A Mixed-Methods Analysis of Adult Continuing Education—Case Study, Egypt Context.”

Although the study questions are important, they require more precise formulation—especially to explicitly reference the target group of adult students in continuing education. For instance, I suggest revising the first research question as follows:

“How do adult students in a continuing education program perceive the flipped classroom experience in general English instruction across the five dimensions of Awidi’s constructivist framework?”

All other research questions should be similarly adjusted. This aligns with what was discussed in the Limitations and Future Research section.

The researchers used authentic and well-documented data, obtained ethical approval (IRB approval #2024-2025-278), and conducted appropriate and varied statistical analyses.

The study is highly significant in its topic, discussion, and recommendations. However, it would be appropriate to rewrite the discussion and recommendations to limit overgeneralization and align the conclusions with the boundaries of the study sample.

I recommend publication. Thank you.

**********

6. PLOS authors have the option to publish the peer review history of their article (what does this mean?). If published, this will include your full peer review and any attached files.

If you choose “no”, your identity will remain anonymous but your review may still be made public.

**Do you want your identity to be public for this peer review?**  For information about this choice, including consent withdrawal, please see our Privacy Policy.

Reviewer #1: **Yes:**  Mohd Elmagzoub Eltahir

Reviewer #2: **Yes:**  Abdellatef Alqawasmi

**********

[NOTE: If reviewer comments were submitted as an attachment file, they will be attached to this email and accessible via the submission site. Please log into your account, locate the manuscript record, and check for the action link "View Attachments". If this link does not appear, there are no attachment files.]

To ensure your figures meet our technical requirements, please review our figure guidelines: https://journals.plos.org/plosone/s/figures

You may also use PLOS’s free figure tool, NAAS, to help you prepare publication quality figures: https://journals.plos.org/plosone/s/figures#loc-tools-for-figure-preparation.

NAAS will assess whether your figures meet our technical requirements by comparing each figure against our figure specifications.

# Author comment: Author response to Decision Letter 1

**DOI:** 10.1371/journal.pone.0353699.r002

13 Nov 2025

Dear Editor and Reviewers,

Thank you for your thoughtful review and constructive comments on our manuscript, [PONE-D-25-50019], “Effectiveness of Flipped Classroom Pedagogy: A Mixed-Methods Analysis of Adult Continuing Education in a Middle Eastern Context.” We appreciate your time and valuable feedback, which has helped us improve the quality of our work. Below, we provide a point-by-point response to each comment, along with details of the changes made.

All reviewers’ comments were accepted, and changes to the manuscript were made accordingly.

Points raised by Reviewer 1, Dr. Mohd Elmagzoub Eltahir

Reviewer Comment Authors’ Response Changes Made

Explicitly link Awidi’s (2006a, 2006b) Model for Improving Student Learning (MISL) to adult-learning and self-efficacy frameworks (e.g., Knowles’ andragogy; Bandura, 1997). Accepted and Addressed A new theoretical synthesis paragraph has been added in Section 2.2 (Literature Review) linking Awidi’s MISL dimensions to Knowles (1974, 1980) and Bandura (1997), with specific examples of how AIR, SAM, PAC, ASF, and KCO correspond to adult learning principles and self-efficacy constructs.

Merge overlapping definitions and repeated citations in Sections 2.1–2.3 (e.g., Bergmann & Sams 2012; Bishop & Verleger 2013; Shao & Liu 2021; Pongpanich et al., 2025) into one concise overview. Accepted and Addressed Sections 2.1–2.3 have been reviewed to streamline and eliminate overlaps in definitions and citations.

Add a Data Availability Statement specifying where anonymized datasets, coding frameworks, or sentiment-analysis scripts can be accessed. Accepted and Addressed A Data Availability Statement has been prepared specifying that anonymized questionnaire datasets, qualitative coding framework, and Python-based sentiment analysis scripts are available upon request from researchers. Due to ethical restrictions protecting participant privacy (IRB approval#2024-2025-278), the statement clarifies procedures for researchers to contact the corresponding author (H. Said, halaalaa@aucegypt.edu) for data access. This statement is submitted in the "Additional Information" section during revision submission.

Points raised by Reviewer 2, Dr. Abdellatef Alqawasmi

Revise the manuscript title to better reflect its scope and context, for example:

“Effectiveness of Flipped Classroom Pedagogy: A Mixed-Methods Analysis of Adult Continuing Education—Case Study, Egypt Context.” Accepted and Addressed The manuscript title was revised to 'Effectiveness of Flipped Classroom Pedagogy: A Mixed-Methods Analysis of Adult Continuing Education—Case Study, Egyptian Context.'

Refine the research questions to explicitly highlight the adult continuing-education population. For example:

“How do adult students in a continuing education program perceive the flipped classroom experience in general English instruction across the five dimensions of Awidi’s constructivist framework?” Accepted and Addressed All research questions have been revised to explicitly reference the adult continuing-education student population, clearly reflecting the study’s scope and context.

Rewrite the discussion and recommendations to avoid overgeneralization and ensure conclusions align closely with the sample’s boundaries. Accepted and Addressed We have comprehensively revised the Discussion and Conclusion sections to ensure precise alignment with our sample’s specific characteristics and boundaries. The revisions include: (1) explicitly identifying our population as adult learners in a continuing education context in the opening paragraph; (2) qualifying all findings and implications with references to the specific sample characteristics (proficiency level A1D, single institution, Egyptian context); (3) adding a prominent scope acknowledgment in the Conclusion stating that findings represent this specific population and should not be generalized to other proficiency levels or regions without additional research; and (4) expanding the Limitations section to further emphasize context-specificity. These revisions ensure readers understand precisely which population and contexts the findings apply to, while maintaining the evidential strength of our mixed-methods analyses.

We trust that the revisions and clarifications provided adequately address the suggestions made by the reviewers. We look forward to receiving the final acceptance on our manuscript and getting the paper published.

Kind regards,

The authors

Attachment Submitted filename: Response to Reviewers for Manuscript [PONE-D-25-50019].docx

# Editor report: Decision Letter 1

**DOI:** 10.1371/journal.pone.0353699.r003

**Authors:** Najeh Alsalhi, Gwo-Jen Hwang

2 Jan 2026

PONE-D-25-50019R1

Effectiveness of Flipped Classroom Pedagogy: A Mixed-Methods Analysis of Adult Continuing Education—Case Study, Egyptian Context

PLOS One

Dear Dr. Said,

Thank you for submitting your manuscript to PLOS ONE. After careful consideration, we feel that it has merit but does not fully meet PLOS ONE’s publication criteria as it currently stands. Therefore, we invite you to submit a revised version of the manuscript that addresses the points raised during the review process.

Please submit your revised manuscript by Feb 16 2026 11:59PM. If you will need more time than this to complete your revisions, please reply to this message or contact the journal office at plosone@plos.org. When you're ready to submit your revision, log on to https://www.editorialmanager.com/pone/ and select the ’submissions Needing Revision' folder to locate your manuscript file.

Please include the following items when submitting your revised manuscript:

- A letter that responds to each point raised by the academic editor and reviewer(s). You should upload this letter as a separate file labeled 'Response to Reviewers'.
- A marked-up copy of your manuscript that highlights changes made to the original version. You should upload this as a separate file labeled 'Revised Manuscript with Track Changes'.
- An unmarked version of your revised paper without tracked changes. You should upload this as a separate file labeled 'Manuscript'.

-->If you would like to make changes to your financial disclosure, please include your updated statement in your cover letter. Guidelines for resubmitting your figure files are available below the reviewer comments at the end of this letter.

If applicable, we recommend that you deposit your laboratory protocols in protocols.io to enhance the reproducibility of your results. Protocols.io assigns your protocol its own identifier (DOI) so that it can be cited independently in the future. For instructions see: https://journals.plos.org/plosone/s/submission-guidelines#loc-laboratory-protocols. Additionally, PLOS ONE offers an option for publishing peer-reviewed Lab Protocol articles, which describe protocols hosted on protocols.io. Read more information on sharing protocols at https://plos.org/protocols?utm_medium=editorial-email&utm_source=authorletters&utm_campaign=protocols.

We look forward to receiving your revised manuscript.

Kind regards,

Gwo-Jen Hwang

Academic Editor

PLOS One

Journal Requirements:

If the reviewer comments include a recommendation to cite specific previously published works, please review and evaluate these publications to determine whether they are relevant and should be cited. There is no requirement to cite these works unless the editor has indicated otherwise.

Please review your reference list to ensure that it is complete and correct. If you have cited papers that have been retracted, please include the rationale for doing so in the manuscript text, or remove these references and replace them with relevant current references. Any changes to the reference list should be mentioned in the rebuttal letter that accompanies your revised manuscript. If you need to cite a retracted article, indicate the article’s retracted status in the References list and also include a citation and full reference for the retraction notice.

Additional Editor Comments:

Although the authors have revised the paper in response to the reviewers’ comments, a few minor issues still need to be addressed before it can be accepted for publication.

1. Please number the research questions, rather than presenting them as multiple paragraphs.

2. Please use complete sentences when referring to the literature. For example, “[30] conducted a controlled university study to assess the impact of a flipped learning approach on general English proficiency among Egyptian EFL undergraduates.” should be revised to “Author(s)’ names [30] conducted a controlled university study to assess the impact of a flipped learning approach on general English proficiency among Egyptian EFL undergraduates.” The authors should check the entire paper to correct this issue consistently.

[Note: HTML markup is below. Please do not edit.]

[NOTE: If reviewer comments were submitted as an attachment file, they will be attached to this email and accessible via the submission site. Please log into your account, locate the manuscript record, and check for the action link "View Attachments". If this link does not appear, there are no attachment files.]

To ensure your figures meet our technical requirements, please review our figure guidelines: https://journals.plos.org/plosone/s/figures

You may also use PLOS’s free figure tool, NAAS, to help you prepare publication quality figures: https://journals.plos.org/plosone/s/figures#loc-tools-for-figure-preparation.

NAAS will assess whether your figures meet our technical requirements by comparing each figure against our figure specifications.

# Author comment: Author response to Decision Letter 2

**DOI:** 10.1371/journal.pone.0353699.r004

7 Jan 2026

Dear Editor,

Thank you for your final review and for your helpful comments on our manuscript [PONE-D-25-50019], “Effectiveness of Flipped Classroom Pedagogy: A Mixed-Methods Analysis of Adult Continuing Education—Case Study, Egyptian Context.” We appreciate your time and guidance in helping us refine our paper for publication.

Below, we provide a point-by-point response to each of your comments, along with the actions taken to address them.

1. Please number the research questions, rather than presenting them as multiple paragraphs: All research questions have been clearly numbered for better readability and structural clarity.

2. Please use complete sentences when referring to the literature. For example, “ conducted a controlled university study…” should read as “Author(s)’ names conducted…” The authors should check the entire paper to correct this issue consistently: We have thoroughly reviewed the entire manuscript to ensure that all literature references are now presented in complete sentences incorporating author names before the citations. This correction has been applied consistently throughout the entire paper.

We trust that these revisions fully address your remaining concerns. We are grateful for your time and consideration, and we look forward to your final decision regarding the acceptance of our manuscript.

Kind regards,

The authors

Attachment Submitted filename: Response to Reviewers.docx

# Peer review: Decision Letter 2

**DOI:** 10.1371/journal.pone.0353699.r005

**Authors:** Najeh Alsalhi, Gwo-Jen Hwang, Hilary Ng

22 May 2026

PONE-D-25-50019R2

Effectiveness of Flipped Classroom Pedagogy: A Mixed-Methods Analysis of Adult Continuing Education—Case Study, Egyptian Context

PLOS One

Dear Dr. Said,

Thank you for submitting your manuscript to PLOS ONE. After careful consideration, we feel that it has merit but does not fully meet PLOS ONE’s publication criteria as it currently stands. Therefore, we invite you to submit a revised version of the manuscript that addresses the points raised during the review process.

Please submit your revised manuscript by Jul 06 2026 11:59PM. If you will need more time than this to complete your revisions, please reply to this message or contact the journal office at plosone@plos.org. When you're ready to submit your revision, log on to https://www.editorialmanager.com/pone/ and select the ’submissions Needing Revision' folder to locate your manuscript file.

Please include the following items when submitting your revised manuscript:

- A letter that responds to each point raised by the academic editor and reviewer(s). You should upload this letter as a separate file labeled 'Response to Reviewers'.
- A marked-up copy of your manuscript that highlights changes made to the original version. You should upload this as a separate file labeled 'Revised Manuscript with Track Changes'.
- An unmarked version of your revised paper without tracked changes. You should upload this as a separate file labeled 'Manuscript'.

If you would like to make changes to your financial disclosure, please include your updated statement in your cover letter. Guidelines for resubmitting your figure files are available below the reviewer comments at the end of this letter.

If applicable, we recommend that you deposit your laboratory protocols in protocols.io to enhance the reproducibility of your results. Protocols.io assigns your protocol its own identifier (DOI) so that it can be cited independently in the future. For instructions see: https://journals.plos.org/plosone/s/submission-guidelines#loc-laboratory-protocols. Additionally, PLOS ONE offers an option for publishing peer-reviewed Lab Protocol articles, which describe protocols hosted on protocols.io. Read more information on sharing protocols at https://plos.org/protocols?utm_medium=editorial-email&utm_source=authorletters&utm_campaign=protocols.

As the corresponding author, your ORCID iD is verified in the submission system and will appear in the published article. PLOS supports the use of ORCID, and we encourage all coauthors to register for an ORCID iD and use it as well. Please encourage your coauthors to verify their ORCID iD within the submission system before final acceptance, as unverified ORCID iDs will not appear in the published article. *Only*  the individual author can complete the verification step; PLOS staff *cannot*  verify ORCID iDs on behalf of authors.

We look forward to receiving your revised manuscript.

Kind regards,

Hilary Ng, Ph.D.

Academic Editor

PLOS One

Journal Requirements:

If the reviewer comments include a recommendation to cite specific previously published works, please review and evaluate these publications to determine whether they are relevant and should be cited. There is no requirement to cite these works unless the editor has indicated otherwise.

Please review your reference list to ensure that it is complete and correct. If you have cited papers that have been retracted, please include the rationale for doing so in the manuscript text, or remove these references and replace them with relevant current references. Any changes to the reference list should be mentioned in the rebuttal letter that accompanies your revised manuscript. If you need to cite a retracted article, indicate the article’s retracted status in the References list and also include a citation and full reference for the retraction notice.

Additional Editor Comments:

PLOS ONE requires publicly accessible underlying data unless a justified exception is provided. The authors should confirm whether a fully anonymised dataset can be deposited in a public repository.

[Note: HTML markup is below. Please do not edit.]

Reviewers' comments:

Reviewer’s Responses to Questions

**Comments to the Author**

1. If the authors have adequately addressed your comments raised in a previous round of review and you feel that this manuscript is now acceptable for publication, you may indicate that here to bypass the “Comments to the Author” section, enter your conflict of interest statement in the “Confidential to Editor” section, and submit your "Accept" recommendation.

Reviewer #3: All comments have been addressed

**********

2. Is the manuscript technically sound, and do the data support the conclusions?

The manuscript must describe a technically sound piece of scientific research with data that supports the conclusions. Experiments must have been conducted rigorously, with appropriate controls, replication, and sample sizes. The conclusions must be drawn appropriately based on the data presented.

Reviewer #3: Yes

**********

3. Has the statistical analysis been performed appropriately and rigorously?

Reviewer #3: Yes

**********

4. Have the authors made all data underlying the findings in their manuscript fully available?

The PLOS Data policy requires authors to make all data underlying the findings described in their manuscript fully available without restriction, with rare exception (please refer to the Data Availability Statement in the manuscript PDF file). The data should be provided as part of the manuscript or its supporting information, or deposited to a public repository. For example, in addition to summary statistics, the data points behind means, medians and variance measures should be available. If there are restrictions on publicly sharing data—e.g. participant privacy or use of data from a third party—those must be specified.

Reviewer #3: No

**********

5. Is the manuscript presented in an intelligible fashion and written in standard English?

PLOS ONE does not copyedit accepted manuscripts, so the language in submitted articles must be clear, correct, and unambiguous. Any typographical or grammatical errors should be corrected at revision, so please note any specific errors here.

Reviewer #3: Yes

**********

6. Review Comments to the Author

Please use the space provided to explain your answers to the questions above. You may also include additional comments for the author, including concerns about dual publication, research ethics, or publication ethics. (Please upload your review as an attachment if it exceeds 20,000 characters)

Reviewer #3: The manuscript offers a methodologically robust mixed-methods study of flipped classroom pedagogy within an adult EFL continuing education framework. The research design aligns with the specified objectives, with properly defined research questions, a cohesive theoretical framework (MISL), and a thoroughly outlined institutional and instructional background. The sample size (n = 297; 72.6% response rate) is sufficient, and the statistical analyses are predominantly robust and well interpreted. The reliability coefficients range from good to outstanding (Cronbach’s α = .721–.929), and the application of multiple regression and MANOVA is suitable for the research enquiries. Assumptions are examined (e.g., Box’s M test, multicollinearity assessed by VIF), and effect sizes are documented. The amalgamation of descriptive statistics, inferential analysis, thematic coding, and sentiment analysis is methodically organised, with findings suitably confined to perceived learning results instead of objective achievement improvements. The manuscript is well-articulated in standard academic English, exhibiting consistent organization and just minor formatting issues that do not impede readability.

The principal issue pertains to data accessibility. The Data Availability Statement specifies that anonymised questionnaire data and scripts are accessible just upon reasonable request due to privacy constraints imposed by the IRB. Although the ethical safeguarding of participant data is crucial, the existing statement fails to adequately satisfy PLOS ONE’s stipulation for unfettered public access to underlying data unless a well-substantiated exception is provided. The authors must specify if a completely anonymised dataset, with the exclusion of potentially identifiable variables, can be submitted to a public repository. No issues are apparent with dual publication, plagiarism, or ethical approval; IRB authorisation and informed consent protocols are thoroughly recorded. The work significantly contributes to the research on adult continuing education, and with improved clarity on data accessibility, it would satisfy publication criteria.

**********

7. PLOS authors have the option to publish the peer review history of their article (what does this mean?). If published, this will include your full peer review and any attached files.

If you choose “no”, your identity will remain anonymous but your review may still be made public.

**Do you want your identity to be public for this peer review?**  For information about this choice, including consent withdrawal, please see our Privacy Policy.

Reviewer #3: No

**********

[NOTE: If reviewer comments were submitted as an attachment file, they will be attached to this email and accessible via the submission site. Please log into your account, locate the manuscript record, and check for the action link "View Attachments". If this link does not appear, there are no attachment files.]

To ensure your figures meet our technical requirements, please review our figure guidelines: https://journals.plos.org/plosone/s/figures

You may also use PLOS’s free figure tool, NAAS, to help you prepare publication quality figures: https://journals.plos.org/plosone/s/figures#loc-tools-for-figure-preparation.

NAAS will assess whether your figures meet our technical requirements by comparing each figure against our figure specifications.

# Author comment: Author response to Decision Letter 3

**DOI:** 10.1371/journal.pone.0353699.r006

28 May 2026

Dear Editor and Reviewer,

Thank you for your final review and for your helpful comments on our manuscript, [PONE-D-25-50019], “Effectiveness of Flipped Classroom Pedagogy: A Mixed-Methods Analysis of Adult Continuing Education—Case Study, Egyptian Context.”

We are grateful to Reviewer #3 for confirming that all prior concerns have been addressed. The reviewer’s positive evaluation of the study’s methodological rigor, statistical analyses, theoretical framework, and written presentation is deeply encouraging and reflects the substantial effort invested across all revision rounds. The sole outstanding issue, data accessibility, has now been fully resolved, as detailed below.

- Comment: PLOS ONE requires publicly accessible underlying data. The authors should confirm whether a fully anonymized dataset can be deposited in a public repository.

- Response: A fully anonymized dataset has been deposited in the Zenodo public repository (https://doi.org/10.5281/zenodo.20363249), with all identifying variables removed. The Data Availability Statement has been updated accordingly.

We are confident this revision fully addresses all outstanding concerns and that the manuscript now meets PLOS ONE’s publication requirements. We sincerely thank the Academic Editor and the Reviewers for their time, constructive guidance, and encouragement throughout this process, and we look forward to a favorable final decision.

Kind regards,

The authors

Attachment Submitted filename: Response to Reviewers for Manuscript.docx

# Editor report: Decision Letter 3

**DOI:** 10.1371/journal.pone.0353699.r007

**Authors:** Najeh Alsalhi, Gwo-Jen Hwang, Hilary Ng, Hilary Ng

28 Jun 2026

Effectiveness of Flipped Classroom Pedagogy: A Mixed-Methods Analysis of Adult Continuing Education—Case Study, Egyptian Context

PONE-D-25-50019R3

Dear Dr. Said,

We’re pleased to inform you that your manuscript has been judged scientifically suitable for publication and will be formally accepted for publication once it meets all outstanding technical requirements.

Within one week, you’ll receive an e-mail detailing the required amendments. When these have been addressed, you’ll receive a formal acceptance letter and your manuscript will be scheduled for publication.

An invoice will be generated when your article is formally accepted. Please note, if your institution has a publishing partnership with PLOS and your article meets the relevant criteria, all or part of your publication costs will be covered. Please make sure your user information is up-to-date by logging into Editorial Manager at Editorial Manager® and clicking the ‘Update My Information' link at the top of the page. For questions related to billing, please contact billing support.

If your institution or institutions have a press office, please notify them about your upcoming paper to help maximize its impact. If they’ll be preparing press materials, please inform our press team as soon as possible -- no later than 48 hours after receiving the formal acceptance. Your manuscript will remain under strict press embargo until 2 pm Eastern Time on the date of publication. For more information, please contact onepress@plos.org.

Kind regards,

Hilary Ng, Ph.D.

Academic Editor

PLOS One

# Editor report: Acceptance letter

**DOI:** 10.1371/journal.pone.0353699.r008

**Authors:** Najeh Alsalhi, Gwo-Jen Hwang, Hilary Ng, Hilary Ng

PONE-D-25-50019R3

PLOS One

Dear Dr. Said,

I'm pleased to inform you that your manuscript has been deemed suitable for publication in PLOS One. Congratulations! Your manuscript is now being handed over to our production team.

At this stage, our production department will prepare your paper for publication. This includes ensuring the following:

* All references, tables, and figures are properly cited

* All relevant supporting information is included in the manuscript submission,

* There are no issues that prevent the paper from being properly typeset

You will receive further instructions from the production team, including instructions on how to review your proof when it is ready. Please keep in mind that we are working through a large volume of accepted articles, so please give us a few days to review your paper and let you know the next and final steps.

Lastly, if your institution or institutions have a press office, please let them know about your upcoming paper now to help maximize its impact. If they'll be preparing press materials, please inform our press team within the next 48 hours. Your manuscript will remain under strict press embargo until 2 pm Eastern Time on the date of publication. For more information, please contact onepress@plos.org.

You will receive an invoice from PLOS for your publication fee after your manuscript has reached the completed accept phase. If you receive an email requesting payment before acceptance or for any other service, this may be a phishing scheme. Learn how to identify phishing emails and protect your accounts at https://explore.plos.org/phishing.

If we can help with anything else, please email us at customercare@plos.org.

Thank you for submitting your work to PLOS ONE and supporting open access.

Kind regards,

PLOS ONE Editorial Office Staff

on behalf of

Dr. Hilary Ng

Academic Editor

PLOS One
